# Bis(2-hy­droxy­benzoato-κ*O*)bis­[3-(4-meth­oxy­phen­yl)-4-(4-methyl­phen­yl)-5-(2-pyrid­yl)-4*H*-1,2,4-triazole-κ^2^ *N* ^1^,*N* ^5^]copper(II) dihydrate

**DOI:** 10.1107/S1600536811016126

**Published:** 2011-05-07

**Authors:** Yan Liu, Zuoxiang Wang, Hai Zhang

**Affiliations:** a School of Chemistry and Engineering, Southeast University, Nanjing 211189, People’s Republic of China

## Abstract

In the title complex, [Cu(C_7_H_5_O_3_)_2_(C_21_H_18_N_4_O)_2_]·2H_2_O, the Cu^II^ atom is located on a centre of inversion and exists in a tetra­gonally distorted octahedral geometry with a CuN_4_O_2_ chromophore. The intra­molecular O—H⋯O hydrogen bond is highly strained due to the mol­ecular geometry and, as a result, is much shorter than expected. Inter­molecular C—H⋯O and C—H⋯O inter­actions are also observed.

## Related literature

For general background to the coordination chemistry of 1,2,4-triazole derivatives, see: Koningsbruggen *et al.* (1997 ▶); Garcia *et al.* (1999 ▶); Klingele & Brooker (2003 ▶); Matsukizono *et al.* (2008 ▶); Suksrichavalit *et al.* (2009 ▶); Rubio *et al.* (2011 ▶). For their biological activity, see: Tozkoparan *et al.* (2000 ▶); Grénman *et al.* (2003) ▶; Alagarsamy *et al.* (2008 ▶); Isloor *et al.* (2009 ▶).
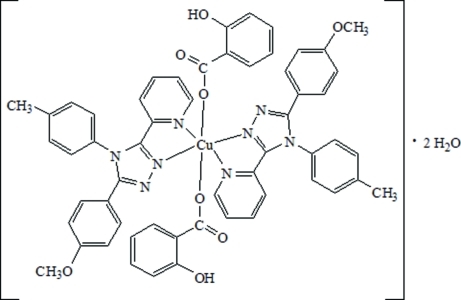


## Experimental

### 

#### Crystal data

[Cu(C_7_H_5_O_3_)_2_(C_21_H_18_N_4_O)_2_]·2H_2_O
*M* *_r_* = 1058.58
Triclinic, 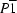

*a* = 8.5933 (12) Å
*b* = 10.6467 (15) Å
*c* = 14.578 (2) Å
α = 103.556 (2)°
β = 91.501 (2)°
γ = 101.843 (2)°
*V* = 1265.1 (3) Å^3^
*Z* = 1
Mo *K*α radiation
μ = 0.50 mm^−1^
*T* = 296 K
0.14 × 0.13 × 0.12 mm

#### Data collection

Bruker APEXII CCD diffractometer
Absorption correction: multi-scan (*SADABS*; Sheldrick, 2003 ▶) *T*_min_ = 0.933, *T*_max_ = 0.942
9025 measured reflections
4413 independent reflections
3891 reflections with *I* > 2σ(*I*)
*R*_int_ = 0.024

#### Refinement

*R*[*F* ^2^ > 2σ(*F* ^2^)] = 0.037
*wR*(*F* ^2^) = 0.101
*S* = 1.08
4413 reflections
349 parameters
H atoms treated by a mixture of independent and constrained refinement
Δρ_max_ = 0.51 e Å^−3^
Δρ_min_ = −0.33 e Å^−3^

### 

Data collection: *APEX2* (Bruker, 2005 ▶); cell refinement: *SAINT* (Bruker, 2005 ▶); data reduction: *SAINT*; program(s) used to solve structure: *SHELXS97* (Sheldrick, 2008 ▶); program(s) used to refine structure: *SHELXL97* (Sheldrick, 2008 ▶); molecular graphics: *SHELXTL* (Sheldrick, 2008 ▶); software used to prepare material for publication: *SHELXTL*.

## Supplementary Material

Crystal structure: contains datablocks I, global. DOI: 10.1107/S1600536811016126/br2165sup1.cif

Structure factors: contains datablocks I. DOI: 10.1107/S1600536811016126/br2165Isup2.hkl

Additional supplementary materials:  crystallographic information; 3D view; checkCIF report

## Figures and Tables

**Table 1:** Hydrogen-bond geometry (Å, °)

*D*—H⋯*A*	*D*—H	H⋯*A*	*D*⋯*A*	*D*—H⋯*A*
O1*W*—H1*F*⋯O2^i^	0.87 (4)	2.02 (4)	2.885 (3)	175 (4)
O1*W*—H1*E*⋯O2	0.78 (4)	2.08 (4)	2.865 (3)	174 (4)
O4—H4⋯O3	0.82	1.79	2.522 (3)	147

Symmetry code: (i) 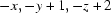.

**Fig. 1.**
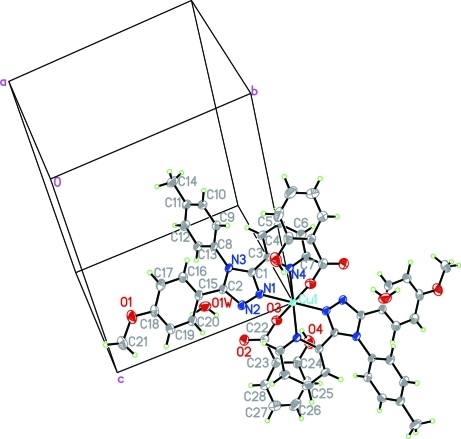
The molecular structure of the title compound with the atomic labelling and the directions of the cell axes. Displacement ellipsoids are shown at 30% probability level.

## supplementary crystallographic information

### Comment

As the 1,2,4-triazole ring posesses strong electron donors, the coordination chemistry of 1,2,4-triazoles as a ligand is widely studied (Koningsbruggen *et al.*, 1997; Garcia *et al.*, 1999; Klingele & Brooker 2003; Matsukizono *et al.*, 2008; Suksrichavalit *et al.*, 2009; Rubio *et al.*, 2011). And some 1,2,4-triazole compounds have biological activities (Tozkoparan *et al.*, 2000; Grénman *et al.*, 2003; Alagarsamy *et al.*, 2008; Isloor *et al.*, 2009). We report here the crystal structure analysis of the title compound. The title copper(II) is surrounded by four N atoms [1.9774 (16)–2.0497 (16) Å] of the two 3-(4-methoxyphenyl)-4-(4-methylphenyl)-5-(2-pyridyl)-4*H*-1,2,4- triazoles in a plane, and two O atoms of the two carboxylate groups interact weakly at axial positions with the copper(II) atom at 2.4322 (16) Å.

There is an intramolecular hydrogen bond of O4-H4···O3, it is highly strained because of the fixed geometry of the molecule. As a result it is much shorter than would otherwise be expected for a bond with this angle.

### Experimental

The title compound was prepared by reaction of 3-(4-methoxyphenyl)-4-(4-methylphenyl)-5-(2-pyridyl)-1,2,4-triazole with copper(II) salicylate in ethanol and water. To a warm solution of 0.684 grams of 3-(4-methoxyphenyl)-4-(4-methylphenyl)-5-(2-pyridyl)-1,2,4-triazole (2mmol) in 20 ml ethanol, 0.338 grams of copper(II) salicylate (1mmol) in 10 ml water was added. The filtrate was left to stand at room temperature for several days. The blue product was collected, and single crystals suitable for X-ray diffraction were selected.

### Refinement

Positional parameters of all the H atoms were calculated geometrically and were allowed to ride on the C, N atoms to which they are bonded, riding with C—H = 0.93 Å (aromatic), 0.96 Å (methyl) or N—H = 0.85 Å, with Uĩso~(H) = 1.2 or 1.5 times U~eq~(C).

### Crystal data

**Table:**

[Cu(C_7_H_5_O_3_)_2_(C_21_H_18_N_4_O)_2_]·2H_2_O	*Z* = 1
*M**_r_* = 1058.58	*F*(000) = 551
Triclinic, *P*1	*D*_x_ = 1.389 Mg m^−^^3^
Hall symbol: -P 1	Mo *K*α radiation, λ = 0.71073 Å
*a* = 8.5933 (12) Å	Cell parameters from 9999 reflections
*b* = 10.6467 (15) Å	θ = 2.4–25.7°
*c* = 14.578 (2) Å	µ = 0.50 mm^−^^1^
α = 103.556 (2)°	*T* = 296 K
β = 91.501 (2)°	Rhombic, blue
γ = 101.843 (2)°	0.14 × 0.13 × 0.12 mm
*V* = 1265.1 (3) Å^3^	

### Data collection

**Table:**

Bruker APEXII CCD diffractometer	4413 independent reflections
Radiation source: fine-focus sealed tube	3891 reflections with *I* > 2σ(*I*)
graphite	*R*_int_ = 0.024
ω scans	θ_max_ = 25.0°, θ_min_ = 1.4°
Absorption correction: multi-scan (*SADABS*; Sheldrick, 2003)	*h* = −10→10
*T*_min_ = 0.933, *T*_max_ = 0.942	*k* = −12→12
9025 measured reflections	*l* = −17→17

### Refinement

**Table:**

Refinement on *F*^2^	Primary atom site location: structure-invariant direct methods
Least-squares matrix: full	Secondary atom site location: difference Fourier map
*R*[*F*^2^ > 2σ(*F*^2^)] = 0.037	Hydrogen site location: inferred from neighbouring sites
*wR*(*F*^2^) = 0.101	H atoms treated by a mixture of independent and constrained refinement
*S* = 1.08	*w* = 1/[σ^2^(*F*_o_^2^) + (0.0507*P*)^2^ + 0.3303*P*] where *P* = (*F*_o_^2^ + 2*F*_c_^2^)/3
4413 reflections	(Δ/σ)_max_ < 0.001
349 parameters	Δρ_max_ = 0.51 e Å^−^^3^
0 restraints	Δρ_min_ = −0.33 e Å^−^^3^

### Special details

**Table:**

Geometry. All e.s.d.'s (except the e.s.d. in the dihedral angle between two l.s. planes) are estimated using the full covariance matrix. The cell e.s.d.'s are taken into account individually in the estimation of e.s.d.'s in distances, angles and torsion angles; correlations between e.s.d.'s in cell parameters are only used when they are defined by crystal symmetry. An approximate (isotropic) treatment of cell e.s.d.'s is used for estimating e.s.d.'s involving l.s. planes.
Refinement. Refinement of *F*^2^ against ALL reflections. The weighted *R*-factor *wR* and goodness of fit *S* are based on *F*^2^, conventional *R*-factors *R* are based on *F*, with *F* set to zero for negative *F*^2^. The threshold expression of *F*^2^ > σ(*F*^2^) is used only for calculating *R*-factors(gt) *etc*. and is not relevant to the choice of reflections for refinement. *R*-factors based on *F*^2^ are statistically about twice as large as those based on *F*, and *R*- factors based on ALL data will be even larger.

### Fractional atomic coordinates and isotropic or equivalent isotropic displacement parameters (Å^2^)

**Table:**

	*x*	*y*	*z*	*U*_iso_*/*U*_eq_	
Cu1	0.0000	1.0000	1.0000	0.03718 (13)	
N1	0.01756 (19)	0.84131 (16)	0.90177 (11)	0.0371 (4)	
O3	0.10103 (18)	0.91416 (16)	1.12470 (12)	0.0527 (4)	
N3	0.15216 (19)	0.73762 (16)	0.79657 (11)	0.0358 (4)	
C8	0.2813 (2)	0.70780 (19)	0.73920 (13)	0.0352 (4)	
N4	0.22652 (19)	1.06205 (16)	0.96344 (11)	0.0361 (4)	
O2	−0.0191 (2)	0.70109 (16)	1.08112 (12)	0.0595 (4)	
C10	0.4232 (3)	0.7231 (2)	0.60350 (15)	0.0441 (5)	
H10	0.4341	0.7492	0.5470	0.053*	
N2	−0.0804 (2)	0.72205 (16)	0.85894 (12)	0.0394 (4)	
C2	0.0031 (2)	0.6597 (2)	0.79623 (14)	0.0374 (5)	
C20	−0.1732 (3)	0.4437 (2)	0.77843 (15)	0.0444 (5)	
H20	−0.1991	0.4751	0.8399	0.053*	
O1	−0.2764 (2)	0.14805 (16)	0.58065 (12)	0.0675 (5)	
C13	0.3854 (3)	0.6414 (2)	0.76912 (15)	0.0434 (5)	
H13	0.3714	0.6120	0.8242	0.052*	
C22	0.0056 (3)	0.8111 (2)	1.13733 (16)	0.0449 (5)	
C15	−0.0608 (2)	0.5241 (2)	0.73933 (14)	0.0390 (5)	
C3	0.2794 (2)	0.97083 (19)	0.89747 (13)	0.0343 (4)	
C9	0.2979 (3)	0.7490 (2)	0.65663 (15)	0.0415 (5)	
H9	0.2259	0.7936	0.6370	0.050*	
C1	0.1555 (2)	0.85032 (19)	0.86417 (13)	0.0351 (4)	
C12	0.5121 (3)	0.6189 (2)	0.71555 (16)	0.0503 (6)	
H12	0.5848	0.5757	0.7361	0.060*	
C23	−0.0796 (3)	0.8294 (2)	1.22638 (15)	0.0449 (5)	
C6	0.4775 (3)	1.2099 (2)	0.97157 (16)	0.0483 (5)	
H6	0.5423	1.2933	0.9965	0.058*	
O4	0.0620 (3)	1.05550 (18)	1.28368 (14)	0.0759 (6)	
H4	0.1018	1.0359	1.2332	0.114*	
C16	−0.0206 (3)	0.4745 (2)	0.64802 (15)	0.0492 (5)	
H16	0.0558	0.5268	0.6208	0.059*	
C7	0.3238 (3)	1.1798 (2)	0.99837 (15)	0.0440 (5)	
H7	0.2871	1.2438	1.0421	0.053*	
C11	0.5333 (3)	0.6588 (2)	0.63257 (16)	0.0449 (5)	
C4	0.4326 (2)	0.9949 (2)	0.86936 (15)	0.0439 (5)	
H4A	0.4676	0.9305	0.8250	0.053*	
C17	−0.0932 (3)	0.3490 (2)	0.59781 (16)	0.0543 (6)	
H17	−0.0652	0.3164	0.5370	0.065*	
C26	−0.2262 (5)	0.8563 (4)	1.3948 (2)	0.0911 (11)	
H26	−0.2734	0.8640	1.4520	0.109*	
C18	−0.2082 (3)	0.2706 (2)	0.63742 (16)	0.0486 (6)	
C19	−0.2477 (3)	0.3182 (2)	0.72827 (16)	0.0477 (5)	
H19	−0.3240	0.2658	0.7554	0.057*	
C5	0.5333 (3)	1.1165 (2)	0.90825 (16)	0.0479 (5)	
H5	0.6377	1.1343	0.8914	0.058*	
C25	−0.1175 (4)	0.9602 (3)	1.38054 (19)	0.0770 (9)	
H25	−0.0921	1.0390	1.4273	0.092*	
C14	0.6730 (3)	0.6345 (3)	0.5758 (2)	0.0704 (8)	
H14A	0.6468	0.6308	0.5107	0.106*	
H14B	0.6971	0.5520	0.5806	0.106*	
H14C	0.7641	0.7051	0.5999	0.106*	
C28	−0.1937 (3)	0.7267 (3)	1.24335 (19)	0.0616 (7)	
H28	−0.2207	0.6470	1.1976	0.074*	
C24	−0.0433 (3)	0.9495 (2)	1.29565 (17)	0.0553 (6)	
C27	−0.2678 (4)	0.7400 (4)	1.3262 (2)	0.0855 (10)	
H27	−0.3457	0.6705	1.3358	0.103*	
C21	−0.3755 (4)	0.0575 (3)	0.6233 (2)	0.0760 (8)	
H21A	−0.3161	0.0441	0.6756	0.114*	
H21B	−0.4118	−0.0255	0.5776	0.114*	
H21C	−0.4657	0.0925	0.6457	0.114*	
O1W	0.1915 (2)	0.5712 (2)	0.96350 (16)	0.0732 (6)	
H1F	0.145 (5)	0.488 (4)	0.949 (3)	0.110*	
H1E	0.139 (5)	0.611 (4)	0.997 (3)	0.110*	

### Atomic displacement parameters (Å^2^)

**Table:**

	*U*^11^	*U*^22^	*U*^33^	*U*^12^	*U*^13^	*U*^23^
Cu1	0.0287 (2)	0.0316 (2)	0.0425 (2)	0.00245 (14)	0.01006 (14)	−0.00540 (14)
N1	0.0317 (9)	0.0324 (9)	0.0411 (9)	0.0041 (7)	0.0062 (7)	−0.0007 (7)
O3	0.0432 (9)	0.0530 (10)	0.0612 (10)	0.0065 (8)	0.0084 (7)	0.0154 (8)
N3	0.0334 (9)	0.0335 (9)	0.0365 (9)	0.0084 (7)	0.0071 (7)	−0.0004 (7)
C8	0.0343 (11)	0.0315 (10)	0.0358 (10)	0.0084 (8)	0.0057 (8)	−0.0012 (8)
N4	0.0301 (9)	0.0340 (9)	0.0387 (9)	0.0050 (7)	0.0063 (7)	−0.0003 (7)
O2	0.0772 (12)	0.0476 (10)	0.0523 (10)	0.0165 (9)	0.0166 (9)	0.0059 (8)
C10	0.0509 (13)	0.0424 (12)	0.0369 (11)	0.0074 (10)	0.0097 (9)	0.0072 (9)
N2	0.0352 (9)	0.0310 (9)	0.0440 (9)	0.0040 (7)	0.0061 (7)	−0.0039 (7)
C2	0.0359 (11)	0.0367 (11)	0.0369 (10)	0.0093 (9)	0.0028 (8)	0.0022 (9)
C20	0.0480 (13)	0.0402 (12)	0.0412 (11)	0.0122 (10)	0.0038 (10)	0.0001 (9)
O1	0.0879 (14)	0.0356 (9)	0.0608 (11)	−0.0029 (9)	−0.0042 (9)	−0.0085 (8)
C13	0.0474 (13)	0.0474 (13)	0.0386 (11)	0.0174 (10)	0.0067 (9)	0.0103 (9)
C22	0.0436 (12)	0.0467 (13)	0.0473 (12)	0.0179 (11)	0.0044 (10)	0.0097 (10)
C15	0.0392 (11)	0.0346 (11)	0.0392 (11)	0.0105 (9)	0.0003 (9)	−0.0004 (9)
C3	0.0324 (10)	0.0351 (11)	0.0334 (10)	0.0084 (8)	0.0052 (8)	0.0033 (8)
C9	0.0442 (12)	0.0388 (12)	0.0428 (11)	0.0145 (10)	0.0033 (9)	0.0079 (9)
C1	0.0333 (11)	0.0344 (11)	0.0353 (10)	0.0097 (9)	0.0061 (8)	0.0018 (8)
C12	0.0464 (13)	0.0582 (15)	0.0534 (13)	0.0273 (11)	0.0085 (10)	0.0130 (11)
C23	0.0452 (13)	0.0469 (13)	0.0460 (12)	0.0195 (10)	0.0053 (10)	0.0097 (10)
C6	0.0369 (12)	0.0461 (13)	0.0499 (13)	−0.0046 (10)	0.0061 (10)	−0.0006 (10)
O4	0.1034 (17)	0.0521 (11)	0.0617 (12)	0.0080 (11)	−0.0054 (11)	0.0021 (9)
C16	0.0552 (14)	0.0407 (13)	0.0436 (12)	0.0049 (11)	0.0054 (10)	−0.0008 (10)
C7	0.0360 (11)	0.0392 (12)	0.0455 (12)	0.0016 (9)	0.0072 (9)	−0.0062 (9)
C11	0.0407 (12)	0.0425 (12)	0.0477 (12)	0.0100 (10)	0.0113 (9)	0.0020 (10)
C4	0.0363 (11)	0.0467 (13)	0.0461 (12)	0.0113 (10)	0.0129 (9)	0.0033 (10)
C17	0.0666 (16)	0.0466 (14)	0.0408 (12)	0.0103 (12)	0.0038 (11)	−0.0053 (10)
C26	0.123 (3)	0.103 (3)	0.074 (2)	0.065 (2)	0.052 (2)	0.036 (2)
C18	0.0582 (14)	0.0337 (12)	0.0475 (13)	0.0103 (10)	−0.0077 (11)	−0.0017 (9)
C19	0.0515 (14)	0.0364 (12)	0.0508 (13)	0.0048 (10)	0.0031 (10)	0.0060 (10)
C5	0.0304 (11)	0.0586 (14)	0.0489 (13)	0.0026 (10)	0.0098 (9)	0.0072 (11)
C25	0.121 (3)	0.072 (2)	0.0498 (15)	0.056 (2)	0.0108 (16)	0.0066 (14)
C14	0.0575 (17)	0.081 (2)	0.0727 (18)	0.0231 (15)	0.0279 (14)	0.0104 (15)
C28	0.0634 (16)	0.0577 (16)	0.0648 (16)	0.0147 (13)	0.0189 (13)	0.0144 (13)
C24	0.0698 (17)	0.0512 (15)	0.0499 (14)	0.0265 (13)	−0.0001 (12)	0.0112 (11)
C27	0.091 (2)	0.089 (2)	0.086 (2)	0.0244 (19)	0.0436 (19)	0.0328 (19)
C21	0.082 (2)	0.0402 (15)	0.090 (2)	−0.0022 (14)	0.0050 (17)	−0.0015 (14)
O1W	0.0569 (12)	0.0694 (13)	0.0760 (13)	−0.0073 (10)	0.0246 (10)	−0.0002 (11)

### Geometric parameters (Å, °)

**Table:**

Cu1—N1	1.9773 (16)	C23—C24	1.402 (3)
Cu1—N4	2.0497 (16)	C6—C5	1.364 (3)
Cu1—O3	2.4322 (16)	C6—C7	1.383 (3)
N1—C1	1.314 (2)	C6—H6	0.9300
N1—N2	1.368 (2)	O4—C24	1.344 (3)
O3—C22	1.282 (3)	O4—H4	0.8200
N3—C1	1.357 (2)	C16—C17	1.374 (3)
N3—C2	1.375 (3)	C16—H16	0.9300
N3—C8	1.449 (2)	C7—H7	0.9300
C8—C13	1.369 (3)	C11—C14	1.508 (3)
C8—C9	1.376 (3)	C4—C5	1.384 (3)
N4—C7	1.334 (3)	C4—H4A	0.9300
N4—C3	1.357 (2)	C17—C18	1.387 (3)
O2—C22	1.236 (3)	C17—H17	0.9300
C10—C9	1.378 (3)	C26—C25	1.354 (5)
C10—C11	1.387 (3)	C26—C27	1.372 (5)
C10—H10	0.9300	C26—H26	0.9300
N2—C2	1.318 (3)	C18—C19	1.380 (3)
C2—C15	1.471 (3)	C19—H19	0.9300
C20—C19	1.378 (3)	C5—H5	0.9300
C20—C15	1.381 (3)	C25—C24	1.398 (4)
C20—H20	0.9300	C25—H25	0.9300
O1—C18	1.373 (3)	C14—H14A	0.9600
O1—C21	1.419 (3)	C14—H14B	0.9600
C13—C12	1.384 (3)	C14—H14C	0.9600
C13—H13	0.9300	C28—C27	1.372 (4)
C22—C23	1.500 (3)	C28—H28	0.9300
C15—C16	1.392 (3)	C27—H27	0.9300
C3—C4	1.381 (3)	C21—H21A	0.9600
C3—C1	1.461 (3)	C21—H21B	0.9600
C9—H9	0.9300	C21—H21C	0.9600
C12—C11	1.378 (3)	O1W—H1F	0.87 (4)
C12—H12	0.9300	O1W—H1E	0.78 (4)
C23—C28	1.385 (3)		
			
N1—Cu1—N4	80.33 (6)	C24—O4—H4	109.5
N1—Cu1—O3	91.91 (6)	C17—C16—C15	120.4 (2)
N4—Cu1—O3	91.28 (6)	C17—C16—H16	119.8
C1—N1—N2	109.27 (16)	C15—C16—H16	119.8
C1—N1—Cu1	114.35 (13)	N4—C7—C6	122.09 (19)
N2—N1—Cu1	136.33 (13)	N4—C7—H7	119.0
C22—O3—Cu1	112.82 (13)	C6—C7—H7	119.0
C1—N3—C2	105.25 (16)	C12—C11—C10	118.0 (2)
C1—N3—C8	126.29 (16)	C12—C11—C14	120.9 (2)
C2—N3—C8	128.45 (16)	C10—C11—C14	121.1 (2)
C13—C8—C9	121.51 (19)	C3—C4—C5	118.87 (19)
C13—C8—N3	119.36 (18)	C3—C4—H4A	120.6
C9—C8—N3	119.12 (17)	C5—C4—H4A	120.6
C7—N4—C3	118.56 (17)	C16—C17—C18	120.2 (2)
C7—N4—Cu1	126.69 (14)	C16—C17—H17	119.9
C3—N4—Cu1	114.75 (13)	C18—C17—H17	119.9
C9—C10—C11	121.3 (2)	C25—C26—C27	120.9 (3)
C9—C10—H10	119.3	C25—C26—H26	119.5
C11—C10—H10	119.3	C27—C26—H26	119.5
C2—N2—N1	106.23 (17)	O1—C18—C19	124.4 (2)
N2—C2—N3	110.19 (17)	O1—C18—C17	115.7 (2)
N2—C2—C15	121.90 (19)	C19—C18—C17	119.8 (2)
N3—C2—C15	127.87 (18)	C20—C19—C18	119.5 (2)
C19—C20—C15	121.4 (2)	C20—C19—H19	120.2
C19—C20—H20	119.3	C18—C19—H19	120.2
C15—C20—H20	119.3	C6—C5—C4	119.2 (2)
C18—O1—C21	117.3 (2)	C6—C5—H5	120.4
C8—C13—C12	118.6 (2)	C4—C5—H5	120.4
C8—C13—H13	120.7	C26—C25—C24	120.2 (3)
C12—C13—H13	120.7	C26—C25—H25	119.9
O2—C22—O3	124.0 (2)	C24—C25—H25	119.9
O2—C22—C23	119.7 (2)	C11—C14—H14A	109.5
O3—C22—C23	116.2 (2)	C11—C14—H14B	109.5
C20—C15—C16	118.57 (19)	H14A—C14—H14B	109.5
C20—C15—C2	117.05 (18)	C11—C14—H14C	109.5
C16—C15—C2	124.3 (2)	H14A—C14—H14C	109.5
N4—C3—C4	121.70 (19)	H14B—C14—H14C	109.5
N4—C3—C1	111.19 (17)	C27—C28—C23	121.4 (3)
C4—C3—C1	127.11 (18)	C27—C28—H28	119.3
C8—C9—C10	118.85 (19)	C23—C28—H28	119.3
C8—C9—H9	120.6	O4—C24—C25	118.4 (3)
C10—C9—H9	120.6	O4—C24—C23	122.1 (2)
N1—C1—N3	109.03 (17)	C25—C24—C23	119.5 (3)
N1—C1—C3	119.30 (17)	C28—C27—C26	119.6 (3)
N3—C1—C3	131.67 (17)	C28—C27—H27	120.2
C11—C12—C13	121.7 (2)	C26—C27—H27	120.2
C11—C12—H12	119.1	O1—C21—H21A	109.5
C13—C12—H12	119.1	O1—C21—H21B	109.5
C28—C23—C24	118.2 (2)	H21A—C21—H21B	109.5
C28—C23—C22	120.7 (2)	O1—C21—H21C	109.5
C24—C23—C22	121.1 (2)	H21A—C21—H21C	109.5
C5—C6—C7	119.5 (2)	H21B—C21—H21C	109.5
C5—C6—H6	120.3	H1F—O1W—H1E	109 (4)
C7—C6—H6	120.3		

### Hydrogen-bond geometry (Å, °)

**Table:**

*D*—H···*A*	*D*—H	H···*A*	*D*···*A*	*D*—H···*A*
O1W—H1F···O2^i^	0.87 (4)	2.02 (4)	2.885 (3)	175 (4)
O1W—H1E···O2	0.78 (4)	2.08 (4)	2.865 (3)	174 (4)
O4—H4···O3	0.82	1.79	2.522 (3)	147

Symmetry codes: (i) −*x*, −*y*+1, −*z*+2.
